# Tactile Feedback in Closed-Loop Control of Myoelectric Hand Grasping: Conveying Information of Multiple Sensors Simultaneously via a Single Feedback Channel

**DOI:** 10.3389/fnins.2020.00348

**Published:** 2020-04-27

**Authors:** Raphael M. Mayer, Ricardo Garcia-Rosas, Alireza Mohammadi, Ying Tan, Gursel Alici, Peter Choong, Denny Oetomo

**Affiliations:** ^1^Human Robotics Laboratory, Department of Mechanical Engineering, The University of Melbourne, Parkville, VIC, Australia; ^2^School of Mechanical, Materials, Mechatronic and Biomedical Engineering, University of Wollongong, Wollongong, NSW, Australia; ^3^ARC Centre of Excellence for Electromaterials Science, Wollongong, NSW, Australia; ^4^Department of Surgery, St. Vincent's Hospital, The University of Melbourne, Parkville, VIC, Australia

**Keywords:** neuroprostheses, sensory feedback restoration, human-robot interaction, tactile feedback, bone conduction

## Abstract

The appropriate sensory information feedback is important for the success of an object grasping and manipulation task. In many scenarios, the need arises for multiple feedback information to be conveyed to a prosthetic hand user simultaneously. The multiple sets of information may either (1) directly contribute to the performance of the grasping or object manipulation task, such as the feedback of the grasping force, or (2) simply form additional independent set(s) of information. In this paper, the efficacy of simultaneously conveying two independent sets of sensor information (the grasp force and a secondary set of information) through a single channel of feedback stimulation (vibrotactile via bone conduction) to the human user in a prosthetic application is investigated. The performance of the grasping task is not dependent to the second set of information in this study. Subject performance in two tasks: regulating the grasp force and identifying the secondary information, were evaluated when provided with either one corresponding information or both sets of feedback information. Visual feedback is involved in the training stage. The proposed approach is validated on human-subject experiments using a vibrotactile transducer worn on the elbow bony landmark (to realize a non-invasive bone conduction interface) carried out in a virtual reality environment to perform a closed-loop object grasping task. The experimental results show that the performance of the human subjects on either task, whilst perceiving two sets of sensory information, is not inferior to that when receiving only one set of corresponding sensory information, demonstrating the potential of conveying a second set of information through a bone conduction interface in an upper limb prosthetic task.

## 1. Introduction

It is well-established that the performance of grasping and object manipulation task relies heavily on the appropriate feedback. This is established in human grasping with or without using prostheses (Childress, 1980; Augurelle et al., 2003) and in robotic grasping algorithms (Dahiya et al., 2009; Shaw-Cortez et al., 2018, 2019). Within prosthetic applications, such feedback allows effective closed-loop control of the prostheses by the human user (Saunders and Vijayakumar, 2011; Antfolk et al., 2013; Markovic et al., 2018; Stephens-Fripp et al., 2018). To date, prosthetic hand users rely on visual and incidental feedback for the closed-loop control of hand prosthesis (Markovic et al., 2018), as explicit feedback mechanisms are not prevalent in commercial prostheses (Cordella et al., 2016). Incidental feedback can be obtained from vibrations transmitted through the socket (Svensson et al., 2017), proprioceptive information from the muscles (Antfolk et al., 2013), sound from the motor (Markovic et al., 2018), or the reaction forces transmitted by the actuating cable in body-powered prostheses (Shehata et al., 2018). Visual feedback has been the baseline feedback mechanism in prosthetic grasping exercises as it is the only feedback available naturally to all commercial hand prostheses (Saunders and Vijayakumar, 2011; Ninu et al., 2014).

It is also established that a combination of feedback information is required—and required simultaneously—for effective grasping and manipulation to be realized. In Westling and Johansson (1984) and Augurelle et al. (2003), it was demonstrated that the maintenance of grip force as a function of the measured load in a vertical lifting scenario is accompanied by their slip detection function. It was argued that in the scenarios of moving a hand-held object, accidental slips rarely occur because “the grip force exceeds the minimal force required” by a safety margin factor. No exceedingly high values of grip force are obtained due to a mechanism measuring the frictional condition using skin mechanoreceptors (Westling and Johansson, 1984). This argues for the use of two sets of information during the operation, namely the feedback of the grip force as well as the information of the object slippage and friction, even if it is to update an internal feed-forward model (Johansson and Westling, 1987). Other examples include an exercise in “sense and explore” where the proprioception information is required along with the tactile information relevant to the object/environment being explored. Information on temperature in addition to the proprioception and tactile could also be needed in specific applications to indicate dangerous temperature, for example when drinking hot beverage using a prosthetic hand—the user may not feel the temperature of the cup until it reaches the lips and causes a burn (Lederman and Klatzky, 1987).

Investigations in the prosthetic literature have so far focused on conveying each independent sensor information to the human user through a single transducer. The feedback is either continuous (Chaubey et al., 2014) or event driven (Clemente et al., 2017) and multiple transducers have been deployed via high density electrotactile arrays (Franceschi et al., 2017). The number of feedback transducers that can be deployed on the human is limited due to the physiology and the available space. Physiologically, the minimum spatial resolution is determined by the two point discrimination that can be discerned on the skin. The minimum spatial resolution is 40 mm for mechanotactile and vibrotactile feedback (on the forearm) and 9 mm for electrotactile feedback (Svensson et al., 2017). An improved result was shown in D'Alonzo et al. (2014), colocating the vibrotactile and electrotactile transducers on the surface of the skin. Spatially, the number of transducers that can be fitted in a transhumeral or transradial socket is limited by the available space within the socket and the contact surface with the residual limb. The limitation of the available stimulation points is even more compelling when using bone conduction for vibrotactile sensation. For osseointegrated implants there is only one rigid abutment point (Clemente et al., 2017; Li and Brånemark, 2017) and for non-invasive bone conduction there are 2–3 usable bony landmarks near the elbow (Mayer et al., 2019). In all these experiments, each sensory information is still conveyed by one dedicated feedback channel.

A few studies have recognized the need for the more efficient use of the feedback channels and proposed the use of multiple sensor information via a single feedback channel. Multiple sets of information have been transmitted in a sequential manner (Ninu et al., 2014), event triggered (Clemente et al., 2016), or representing only a discrete combination of the information from two sensors (Choi et al., 2016, 2017). Time sequential (Ninu et al., 2014) or event triggered feedback (Clemente et al., 2016) can be used for tasks or events where the need for each sensing information can be decoupled over the subsequent events, therefore do not address the need described above for simultaneous feedback information.

Of the many facets of the challenges in closing the prosthetic control loop through the provision of effective feedback, we seek in this paper to improve the information density that can be conveyed through a single stimulation transducer to deliver multiple sets of feedback information simultaneously to the prosthetic user. Specifically, the amplitude and the frequency of the stimulus signal are used to convey different information. This concept was observed in Dosen et al. (2016), where a vibrotactile transducer was designed to produce independent control of the amplitude and the frequency of the stimulation signal. It was reported that a psychophysical experiment on four healthy subjects found 400 stimulation settings (a combination of amplitude and frequency of the stimulus signal—each termed a “vixel”) distinguishable by the subjects.

In this paper, the efficacy of this concept is further investigated on a closed-loop operation of a hand prosthesis in virtual reality. One set of information, the grasp force, is used in the closed-loop application, providing sensory feedback on the grasp force regulated by the motor input via surface electromyography (sEMG). The second set provides an additional secondary information. Note that a closed-loop operation differs from psychometric evaluation as the sensory excitation is a function of the voluntary user effort in the given task. This study is investigated within the context of non-invasive bone conduction interface, where the need for higher information bandwidth is compelling due to the spatial limitations in the placement of feedback transducer to the human user. It should be noted that the purpose of the study in this paper is to establish the ability for a second piece of information to be perceived. Once this is established, the second set of information may be used to (1) perceive an independent set of information, such as the temperature of the object grasped, or (2) improve the performance of the primary task with additional information. In this paper, the second set of information is not expected to improve the performance of the primary task, which is the closed loop object grasping task.

It was found that the human subjects were able to discern the two sets of information even when applied simultaneously. The baseline for comparison is the case where only one set of sensor information was directly conveyed as feedback to the human user. Comparing the proposed technique to the baseline, a comparable performance in regulating the grasp force of the prosthesis (accuracy and repeatability) and in correctly identifying the secondary information (low, medium, or high) was achieved.

## 2. Conveying Multi-Sensor Information via Fewer Feedback Channels

We define the sensor information as **y** ∈ *R*^*N*^, where *N* is the number of independent sets of sensor information, where the measurements can be continuous-time signals or discrete events. The feedback stimulation to the prosthetic user is defined as **x** ∈ *R*^*M*^, where *M* is the number of channels (transducers) employed to provide the feedback stimulation. The scenario being addressed in this paper is that where *N* > *M*. The relationship between measurement **y** and the feedback stimulation *x* can be written as

(1) $$\begin{array}{l} \text{x}=\phi \left(\text{y}\right) \end{array}$$

where ϕ : *R*^*N*^ → *R*^*M*^.

### 2.1. Sensor Information y

Four major sensing modalities are generally present in the upper limbs: touch, proprioception, pain, and temperature. The touch modality is further made up of a combination of information: contact, normal and shear force/pressure, vibration, and texture (Antfolk et al., 2013). To achieve a robust execution of grasping and object manipulation task, only a subset of these sensing modalities are used as feedback. Recent studies have further isolated the types of feedback modalities and information that would be pertinent to an effective object grasping and manipulation, such as grip force and skin-object friction force (de Freitasnzo et al., 2009; Ninu et al., 2014). Furthermore, literature has explicitly determined that such combination of feedback information is required simultaneously for an effective grasping and manipulation (Westling and Johansson, 1984; Augurelle et al., 2003). In the context of an upper limb prosthesis, it is possible to equip a prosthetic hand with a large number of sensors (Kim et al., 2014; Mohammadi et al., 2019) so that *N* > *M*. It should be noted that the *N* sets of independent information can be constructed out of any number of sensing modalities, such as force sensing, grasp velocity sensing, tactile information e.g., for object roughness. It may even contain estimated quantities that cannot be directly measured by sensors, for example: object stiffness may require the measurements of contact force and displacement.

### 2.2. Feedback Stimulation x

The state of the art of non-invasive feedback in prosthetic technology generally utilizes electrotactile (ET), vibrotactile (VT), and mechanotactile (MT) modalities, placed in contact with the skin as a way to deliver the sensation (Stephens-Fripp et al., 2019). More novel feedback mechanisms have also been explored, such as using augmented reality (Markovic et al., 2014). Of these modalities, ET and VT present the challenges of a varying stimulation perception with location of application, VT also presents the challenge where its perception is static-force dependent (i.e., it depends on how hard the VT transducer is pressed against the skin) while MT is often bulky, with high power consumption (Svensson et al., 2017; Stephens-Fripp et al., 2018).

It was shown, however, that VT applied over bony landmarks does not suffer from the static force dependency (Mayer et al., 2018), is compact and does not suffer from high power consumption (Mayer et al., 2019). It does, however, restrict the locations that this technique can be applied to on the upper limb, as there are relatively fewer bony landmarks on the upper limb than skin surface. A psychophysical evaluation in Mayer et al. (2019) demonstrates comparable results in non-invasive vibrotactile feedback on the bone to the invasive (osseointegrated) study in Clemente et al. (2017). It is highlighted that personalization is required for the perception threshold in order to be used as an interface. A higher sensitivity has been reported for frequencies in the range of 100–200 Hz where lower stimulation forces are required. This allows the use of more compact transducers with lower power consumption (Mayer et al., 2019).

### 2.3. Specific Sensor Information and Feedback Stimulation Utilized

In order to demonstrate the concept of conveying multi-sensor information via fewer feedback channels, this paper uses one feedback channel to convey two sets of independent sensor information, namely the grasp force *f*_*g*_ and a secondary information *s*, which could be e.g., skin-object friction, temperature. That is,

(2) $$\begin{array}{l} \text{y}=\left[\begin{array}{c} f_{g}\\ s \end{array}\right]\in R^{2}, \end{array}$$

where *f*_*g*_ represents a continuous-time signal of the grasp force and *s* is a discrete class of the secondary information. The primary information, the grasp force, is used as a feedback to the task of regulating the object grasp force. The secondary information does not directly contribute to the task of regulating the grasp force.

VT via bone conduction is selected as the feedback stimulation, applied on the elbow bony landmark. The sinusoidal waveform applied as the vibrotactile stimulus is:

(3) $$\begin{array}{l} x\left(t\right)=a\left(t\right)\text{ sin}\left(2\pi f\left(t\right)t\right), \end{array}$$

where the amplitude *a*(*t*) is modulated as a linear function of the continuous-time grasp force signal *f*_*g*_(*t*):

(4) $$\begin{array}{l} a\left(t\right)=a_{0}+k_{a}f_{g}\left(t\right). \end{array}$$

while the frequency *f*(*t*) is modulated as a linear function of the secondary information *s*(*t*)

(5) $$\begin{array}{l} f\left(t\right)=f_{0}+k_{s}s\left(t\right), \end{array}$$

where *s*(*t*) ∈ {*S*1, *S*2, *S*3} is a discrete set describing the secondary information at time *t*. The offset *a*_0_ and *f*_0_ denote the minimum amplitude and frequency detectable by human bone conduction perception. The constants *k*_*a*_ and *k*_*s*_ are positive.

## 3. Methodology

**Figure 1:**
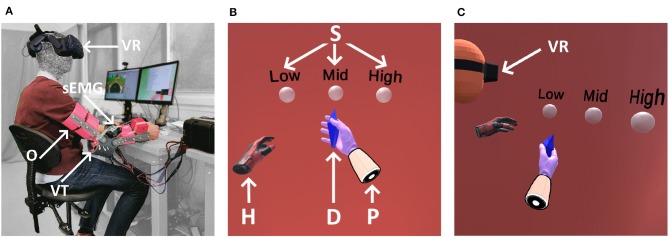
The setup of the grasp task in virtual reality is shown in **(A)** where the subject is seated with the arm placed in the orthoses (O) onto the table. The vibrotactile transducer (VT) is mounted onto the ulnar olecranon; the EMG electrodes (sEMG) onto the forearm and the virtual reality headset (VR) placed on the subjects head. The subjects first person view in virtual reality in **(B)** shows the prosthetic (P); the grasped object (D); the non-dominant hand (H) for commands as activating/deactivating EMG or touching the sphere (S) for reporting the secondary information class and to advance to the next task; **(C)** shows the top person view of the virtual reality setup.

The proposed approach is validated in a human-subject experiment using a VT transducer worn on the elbow bony landmark to provide the feedback and a virtual reality based environment to simulate the grasping task, as shown in Figure 1A. This experiment seeks to verify that subjects can differentiate two encoded sensory information conveyed via one bone conduction channel. This is done by firstly, comparing the performance of the proposed approach against the baseline of carrying out the same task with only one set of information conveyed through the feedback channel. Secondly, the performance with and without the addition of visual feedback was compared.

The experiment consists of three parts:

1. A pre-evaluation of the psychophysics of the interface;
2. Obtaining the bone conduction perception threshold at the ulnar olecranon for individual subjects;
3. Evaluating the performance of the human subject in the task of grasping within a virtual reality environment.

The experiment was conducted on 10 able-bodied subjects (2 female, 8 male; age 28.7 ± 4 years). Informed consent was received from all subjects in the study. The experimental procedure was approved by the University of Melbourne Human Research Ethics Committee, project numbers 1852875.2 and 1750711.1.

### 3.1. Psychophysics

This subsection performs the psychophysical evaluation of the bone conduction interface as sensory feedback. This is done to ensure that subjects can discriminate between the given stimulation frequencies and amplitudes chosen in the later for the Grasp Force Regulation and Secondary Information Classification Task (see section 3.3). Therefore the minimum noticeable difference for subjects, later referred to as “just noticeable difference” (JND), is obtained to quantify the capabilities of the bone conduction interface in frequency and amplitude domain.

#### 3.1.1. Setup

##### 3.1.1.1. Orthosis

A custom elbow orthosis with adjustable bone conduction transducers was fitted to the subjects dominant hand for the experiment as shown in Figure 1A. The orthosis (O) was fixed to the upper and lower arm of the subject through adjustable velcro straps. The vibrotactile transducer (VT) position was adjusted by a breadboard-style variable mounting in order to align and be in contact with the ulnar olecranon, which is the proximal end of the ulna located at the elbow. The VT is adjusted using two screws to ensure good contact with the bony landmark. The orthosis is placed on the desk (see Figure 1A), and kept static during the experiments.

##### 3.1.1.2. Bone conduction

The setup consists of a B81 transducer (RadioEar Corporation, USA), calibrated using an Artificial Mastoid Type 4930 (Brüel & Kjære, Denmark) at the static force of 5.4 N. The stimulation signals were updated at 90 Hz and amplified using a 15 W Public Address amplifier Type A4017 (Redback Inc., Australia) having a suitable 4 − 16Ω output to drive the 8Ω B81 transducers and a suitable low harmonic distortion of < 3% at 1 kHz. Calibrated force sensitive resistor (FSR) (Interlink Electronics 402 Round Short Tail), placed between the transducer and the mounting plate, were used to measure the applied force using a force sensitive area of *A* = 1.33*cm*^2^. The calibration was done using three different weights [0.2, 0.5, 0.7] kg measuring five repetitions and applying a linear interpolation to obtain the force/voltage relationship. The achieved force/voltage relationship has a variance of 5.4±0.37N. The stimulation signal was generated using a National Instruments NI USB-6343 connected to a Windows Surface Book 2 (Intel Core i7-8, 16GB RAM, Windows 10™) as control unit. A MATLAB® GUI was used to guide the user through the psychophysics and perception threshold experiment. The computer was connected via a Wi-Fi hotspot through a UDP connection to the head mounted virtual reality system for the experiment tasks.

#### 3.1.2. Protocol

It is noted that the JND of frequency (JND_f_) as well as the JND of amplitude (JND_a_) are different for each person (Dosen et al., 2016). Therefore, a sample of five subjects are employed to evaluate JND_f_ and JND_a_ to show that the subjects can discriminate between the given stimulation frequencies and amplitudes. The JND_f_ is measured for three frequencies *f*_*ref*_ ∈ [100, 400, 750] Hz and three amplitudes *a*_*ref*_ ∈ [0.1, 0.3, 0.5]*V*, giving the permutation of the nine different combinations. For each combination, a standard two-interval forced-choice (2IFC) threshold procedure is used. For the 2IFC, the reference stimulus *f*_*ref*_ is selected out of the three predetermined frequencies and the target stimulus *f*_*t*_ was varied in a stochastic approximation staircase (SAS) manner, where the variation is based on the report of the subjects of the perceived stimulus (Clemente et al., 2017). Therefore,

(6) $$\begin{array}{l} f_{t_{n+1}}=f_{t_{n}}-\frac{1.5f_{ref}}{\left(2+m\right)}\left(Z_{n}-0.85\right), \end{array}$$

where *f*_*t*_*n*__ is the target stimulus during the previous trial, *f*_*t*_*n*+1__ is the upcoming trial, *m* is the number of reversals showing how many times the answers change from wrong to right, *Z*_*n*_ is set to 1 for correct answer and 0 for an incorrect answer, and *f*_*t*_*n*__ is initialized with 1.5 times the reference stimulus. The trials are stopped after 50 iterations and the value *f*_*t*_51__ for the 51st trial is taken as the perception threshold (Clemente et al., 2017; Mayer et al., 2019).

The JND_a_ is obtained similar to the JND_f_ where the target amplitude *a*_*t*_*n*+1__ is now varied in a SAS manner and the reference stimulus *a*_*ref*_ is chosen out of the given amplitudes.

### 3.2. Perception Threshold

The objective of this subsection is to determine the minimum stimulation amplitude *a*_0_ from which subjects could perceive a given stimulation frequency *f*. This will be referred to as “perception threshold” henceforth. For any given frequency, the amplitude thresholds change and are different for each person, thus it is necessary to be identified (Mayer et al., 2019).

#### 3.2.1. Setup

The same setup as for the psychophysical evaluation was used which is explained in section 3.1.1.

#### 3.2.2. Protocol

The perception threshold is obtained using a method of adjustment test (Kingdom and Prins, 2016, Chapter 3). The subjects are presented *n* = 10 times with each frequency *f* ∈ [100, 200, 400, 750, 1500, 3000, 6000] Hz. At each iteration, the amplitude is adjusted by the subject to the lowest perceived stimulation. The subject can adjust the amplitude in small Δ*U*_*small*_ = 0.005*V* and large Δ*U*_*large*_ = 0.05*V* increments. The frequencies were presented in a randomized order.

The obtained perception threshold value *a*_0_ for each subject is set in the bone conduction stimulation signal (Equation 4). The experiment then proceeded to the virtual reality based grasping tasks.

### 3.3. Grasp Force Regulation and Object Classification

Subjects were asked to perform a set of grasp force regulation and secondary information classification tasks with a virtual prosthetic hand. The tasks involved regulating the grasp force of the virtual reality prosthetic hand through the use of a sEMG-based control interface and classifying the secondary information. Different combinations of feedback modalities [visual feedback (V), grasp force (F), and the secondary information (S)] were presented, as shown in Table 1.

**Table 1:** Experimental cases tested: encoding two sets of information onto the amplitude and frequency of the vibrotactile stimulation as the feedback to the subject through bone conduction.

	**Task**	**V**	**F**	**S**
VF	Grasp force	x	x	
VS	Secondary information	x		x
VFS	Mixed	x	x	x
F	Grasp force		x	
S	Secondary information			x
FS	Mixed		x	x

*The role of visual feedback is also compared as a baseline*.

*The efficacy of this concept is further investigated on a closed-loop operation of a hand prosthesis*.

Three grasping tasks were tested in each group (Table 1). “Grasp Force Regulation Task” consisted purely of applying a grasp force to an object in-hand, this task is detailed in section 3.3.3.1. “Secondary Information Classification Task” consisted of classifying the secondary information, with no grasp force involved, this task is detailed in section 3.3.2.2. “Mixed Task” was a combination of “Grasp Force Regulation Task” and “Secondary Information Classification Task,” where subjects required to apply a given grasp force and classify the secondary information, this task is detailed in section 3.3.2.3. Tasks VF and VS were considered as training for the users to familiarize themselves with the sEMG control interface and the feedback. Tasks VFS, F, S, and FS were used to show if subjects can differentiate multiple sensory feedback encoded in one channel with and without visual feedback. The tasks are detailed in the following subsections.

#### 3.3.1. Setup

##### 3.3.1.1. Orthoses and bone conduction

The same setup as for the psychophysical evaluation was used, as explained in section 3.1.1.

##### 3.3.1.2. sEMG

MyoWare sensors with Ag-AgCl electrodes were used for sEMG data gathering. Data gathering and virtual reality update were performed at 90 Hz.

##### 3.3.1.3. Virtual reality

The virtual reality component of the experiment was performed on an HTC Vive Pro HMD with the application developed in Unity3D. The experimental platform runs on an Intel Core i7-8700K processor at 3.7 GHz, with 32 GB RAM, and GeForce GTX 1080Ti video card with 11 GB GDDR5. An HTC Vive Controller was used for tracking the non-dominant hand of the subject and to interact with the virtual reality application. The subjects report on the secondary information and navigate through the experiment with the non-dominant hand. An HTC Vive Tracker was used to determine the location of the dominant hand of the subject to determine the location of the virtual prosthesis. The application used for the experiment can be downloaded from https://github.com/Rigaro/VRProEP.

An average time latency of a touch event generated in virtual reality and the activation of the feedback stimulus of *t*_*latency*_ = 66 ms was estimated by measuring the single time latency's involved. The total delay results from the time delay of sending a command from the virtual reality setup via a UDP connection to the stimulation control unit *t*_*UDP*_ = 65 ms (measured) and the delay of sending the stimulation command to the NI USB-6343 *t*_*NI*_ = 1 ms (datasheet).

#### 3.3.2. Protocol

Subjects performed a set of grasping and secondary information classification tasks in the virtual reality environment. The tasks were separated into two blocks (see Table 1) with a 2 min break between them. An HTC Vive Pro Head Mounted Display (HMD) was used to display the virtual reality environment to subjects. The virtual reality set-up is shown in Figure 1A while the subject's first person view in virtual reality is shown in Figure 1B and a top person view in Figure 1C. A Vive Controller was held by the subject on their non-dominant hand and was used to enable the EMG interface by a button press and to select the secondary information class in the classification task. A standard dual-site differential surface EMG proportional prosthetic interface was used to command the prosthetic hand closing velocity (Fougner et al., 2012). Muscle activation was gathered using sEMG electrodes placed on the forearm targeting wrist flexor and extensor muscles for hand closing and opening, respectively.

##### 3.3.2.1. Grasp force regulation task

In the grasp force task, subjects were asked to use the sEMG control interface to regulate the grasp force to grip objects with a certain grasp force level. A fixed stimulation frequency was used, in line with the result of the psychophysical evaluation, while the amplitude *a*(*t*) is used to provide feedback on the grasp force produced by the human subject as determined by Equation (4).

The grasp force *f*_*g*_ was calculated from the sEMG signal magnitude. Therefore, the sEMG signal magnitude *u*_*EMG*_ is integrated in a recursive discrete manner, as given by

(7) $$\begin{array}{r} -100\leq u_{EMG}\left(k\right)\leq 100,\\ f_{g}\left(k+1\right)=f_{g}\left(k\right)+\Delta f_{g}u_{EMG}\left(k\right),\\ 0\leq f_{g}\left(k+1\right)\leq 1, \end{array}$$

where *u*_*EMG*_(*k*) is the sEMG input amplification adjusted per subject to range from [−100, 100]; Δ*f*_*g*_ = 0.005 is the scaling factor to convert sEMG signal magnitude to a force rate of change. The grasp force *f*_*g*_ is bounded to [0, 1]. The recursion is updated at 90 Hz.

The grasp tasks were grouped in two parts (see Table 1). In the first part (VF), visual feedback related to the grasp force was given to the subjects and is considered as training. The visual feedback consisted of the grasped object changing color in a gradient depending on the applied grasp force *f*_*g*_(*k*). In the second part (F), no visual feedback was provided. Three different target grasp force levels were used for the task and each was repeated five times in a randomized manner. The target grasp force levels were [0.3, 0.5, 0.8]. The object starting color represented the target grasp force level, however, subjects did not explicitly know the exact target force.

##### 3.3.2.2. Secondary information classification task

In the secondary information classification task, subjects were asked to report on which of the three different classes they perceived by touching one of three spheres in front of them representing each of the classes. The classes (*s*) were low, mid, and high, which translated to the following frequencies [100, 400, 750] Hz. The grasp force was set to constant at *f*_*g*_ = 0.8 resulting in a constant amplitude *a*(*t*) in the feedback stimulus to the subject for this task. In other words, it is not regulated based on the subject sEMG involvement. Each class was presented 5 times in a randomized manner. The secondary information classification tasks were grouped in two parts (see Table 1). In the first part (VS), visual feedback related to the correct class was shown to the subject through the color of the classification spheres whilst presenting the stimuli and is therefore considered as training. In the second part (S), no visual feedback was provided.

##### 3.3.2.3. Mixed task

The mixed task combines both the grasp force regulation and secondary information classification tasks simultaneously, such that the grasp force regulation had to be executed and the subjects were then asked to report on which secondary information class they perceived. This means that the stimulation provided to the subjects had the grasp force *f*_*g*_(*k*) encoded in its amplitude *a*(*t*) and the secondary information class *s* encoded in its frequency *f*, simultaneously. A permutation of all force levels and secondary information classes was presented and each combination was repeated five times in a randomized manner. Force levels are [0.3, 0.5, 0.8] and secondary information classes are [100, 400, 750] Hz. The mixed tasks were grouped in two parts (see Table 1). In the first part (VFS), visual feedback related to the grasp force was given to the subjects. No visual feedback was given for the secondary information feedback. In the second part (FS), no visual feedback was provided.

#### 3.3.3. Data Gathering and Performance Measure

The grasp force *f*_*g*_ (as calculated in Equation 7) and the actual sEMG activation levels were continuously recorded for all trials for the duration of each task, along with the desired force target. The subject's answer for the Secondary Information Classification Task was recorded for tasks “Secondary Information Classification Task” and “Mixed Task,” along with the correct class.

The following performance measures were used:

**Normalized Grasp Force:** is the normalized grasp force *f*_*g*_(*k*_*f*_) at the time *k*_*f*_, where *k*_*f*_ is the time the subjects finalize the force adjustment by disabling the EMG interface by a button press. The mean and standard deviation is calculated over the repetitions of each task regulating the grasp force and represents the accuracy and repeatability of the grasp force regulation exercise.
**Secondary Information Classification Rate:** The rate with which the subject identifies the correct secondary information class was used as the performance measure in the secondary information classification task.

The achieved results of perception threshold, secondary information classification rate and normalized grasp force are visually presented using boxplots, showing the median, 25th and 75th percentiles and the whiskers indicating the most extreme points not considered outliers. Any outliers are plotted using the “+” symbol.

For statistical analysis a non-parametric ANOVA like analysis, specifically a Friedman test was applied (Daniel, 1990) as an ANOVA due to non normal distributed data (Shapiro–Wilk test) was not suitable. This was followed up by a *post-hoc* analysis via Wilcoxon signed rank test (Wilcoxon, 1945). The obtained *p* values are given as well as the statistical significance indicated in the plots.

## 4. Results

Before using the VT bone conduction feedback interface, a pre-evaluation of the psychophysics of this interface is conducted and the perception threshold of each subject determined.

### 4.1. Psychophysics

**Figure 2:**
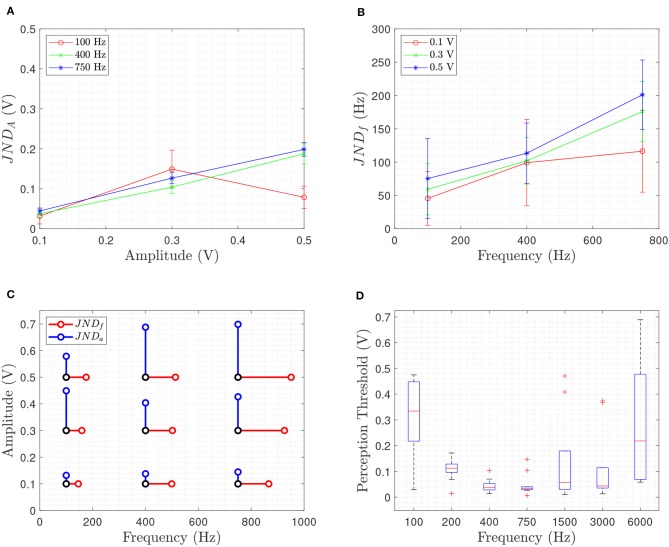
Results of the psychophysical evaluation of five subjects show the mean and standard deviation of the **(A)** JND_a_ giving the amplitude resolution for three different frequencies at three different amplitudes and **(B)** JND_f_ giving the frequency resolution for three different amplitudes at three different frequencies while **(C)** shows a summary plot of the obtained mean value of JND_a_ (blue) and JND_f_ (red) at each reference stimulus (black). In **(D)**, the identified perception threshold value *a*_0_ at the frequencies [100, 200, 400, 750, 1500, 3000, 6000] Hz for 10 subjects, is shown.

Figures 2A,B show the obtained mean and standard deviation for the JND_a_ and JND_f_. In Figure 2C, the mean of both JND_a_ and JND_f_ are plotted together to show the resolution of the proposed interface. The black dots in Figure 2C denote the reference stimulus of the SAS approach and the red and blue dots show the obtained mean value of the JND. Therefore, this plot shows the next closest noticeable stimulation point (frequency or amplitude).

The results in Figure 2C show that the JND_a_ is the smallest for lower frequencies except at 100 Hz and 0.3 V reference stimulus. Hence, the fixed frequency of the grasp force regulation task, as discussed in section 3.3.2, was set to 100 Hz since subjects had the best amplitude discrimination. Comparing the results obtained for VT on skin in Dosen et al. (2016), Figures 2A,B show similar behavior where the JND is increasing linearly with increasing amplitude and frequency. The lower value for JND_a_ at 100 Hz indicates better sensitivity at lower frequencies for higher stimulation amplitudes in case of bone conduction.

### 4.2. Perception Threshold

Before applying VT bone conduction feedback, the lowest perceived stimulation at the given frequencies was found using a method of adjustment. This threshold *a*_0_ was used in Equation (4) to fit the linear relation. The maximum was set to half of the maximum transducer voltage of 0.5 V. Figure 2D shows the obtained perception threshold for all subjects.

### 4.3. Grasp and Object Classification

In the following subsections, the performance of the Mixed Task, representing the proposed concept of conveying two sets of information simultaneously via one feedback channel to the human subject, is compared to the baseline performance of the Grasp Force Regulation Task and the Secondary Information Classification Task, using the defined performance measures.

#### 4.3.1. Secondary Information Classification Rate

**Figure 3:**
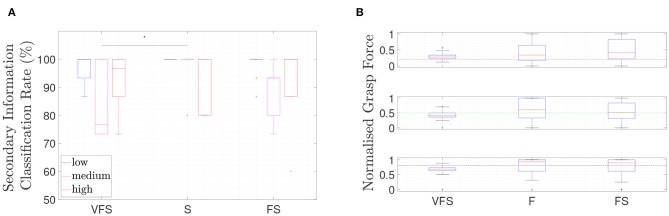
Boxplots of the obtained results of the **(A)** secondary information classification task, subdivided into the three secondary information classes, and in **(B)** the achieved grasp force, subdivide into the three target grasp force levels, is shown for 10 subjects. *Asterisk indicates statistical difference by *post-hoc* analysis *p* < 0.05.

The obtained secondary information classification rates are shown in the boxplot of Figure 3A for the VFS, S, and FS tasks. VS and VF are the training tasks and therefore the obtained data is not considered in the plots. In VS, the subjects received visual feedback for the correct answer in order to learn how to interpret the secondary information feedback and therefore reached 100% secondary information classification rate. In S, only secondary information feedback via bone conduction is provided without visual feedback. In FS, the grasp force level has to be adjusted and the correct secondary information class chosen afterwards, with both grasp force and secondary information feedback provided simultaneously via the bone conduction mechanism.

A mean secondary information classification rate of 86.22±18.17% for VFS (visual, force, and secondary information feedback), 92.00±16.57% for S (secondary information feedback) and 89.11±16.16% for FS (force and secondary information feedback) has been observed. The mean secondary information classification rate and standard deviation for each class (low, medium, high) for the three different tasks are given in Table 2 and the boxplot shown in Figure 3A. A Friedman test (VFS, S, FS) for secondary information classification rate resulted in a statistical significance for the medium secondary information class classification (see Table 3).

**Table 2:** Shows the mean and standard deviation of the obtained results for secondary information classification rate and normalized grasp force for the 4 tasks for 10 subjects.

	**Level**	***VFS***	***F***	***S***	***FS***
Secondary information	Low	97.33 ± 4.66	−	100.00 ± 0.00	98.00 ± 4.50
Classification rate (%)	Medium	76.67 ± 25.19	−	90.00 ± 25.38	84.67 ± 19.89
	High	84.67 ± 30.96	−	86.00 ± 25.03	84.67 ± 28.12
Normalized grasp force	0.2	0.29 ± 0.08	0.45 ± 0.33	−	0.50 ± 0.33
	0.5	0.43 ± 0.11	0.60 ± 0.35	−	0.56 ± 0.29
	0.8	0.68 ± 0.09	0.80 ± 0.24	−	0.80 ± 0.24

**Table 3:** The *p*-values of the Friedman test for the secondary information classification rate for the three different classes.

	**Secondary information class**		
	**Low**	**Medium**	**High**
*p* value	0.174	0.031	0.717

*A significance level of p < 0.05 was used*.

For low and high secondary information class, no statistical significance could be found, suggesting the data is compatible with all groups having the same distribution. For medium secondary information class a Wilcoxon signed rank test is applied as *post-hoc* test and results are shown in Table 4. A statistical significance could be found for VFS vs. S, but not for VFS vs. FS and S vs. FS suggesting the data is compatible with all groups having the same distribution.

**Table 4:** The *p*-values of the *post-hoc* Wilcoxon signed rank test for medium class of the mean secondary information classification rate.

	**Task**		
	***VFS* vs.S**	***VFS* vs.FS**	***S* vs. *FS***
*p* value	0.024	0.062	0.255

*A significance level of p < 0.05 was used*.

#### 4.3.2. Normalized Grasp Force

Figure 3B shows the boxplot of the achieved grasp force by the subjects during VFS, F, and FS. In VF, the subjects received visual feedback for the applied grasp force to learn how to associate grasp force to visual feedback as well as tactile feedback. In all cases, grasp force feedback is present, while visual feedback is present only in VFS (see Table 1). The result of each force level and each trial is given in Table 2.

The obtained results for the Friedman Test (VFS, F, FS) of all force levels are shown in Table 5 and no statistical significance could be found suggesting the data is compatible with all groups having the same distribution.

**Table 5:** The *p*-values of the Friedman test for the Normalized Grasp Force for the different target levels.

	**Target grasp force**		
	**0.2**	**0.5**	**0.8**
*p* value	0.150	0.407	0.150

*A significance level of p < 0.05 was used*.

## 5. Discussion

### 5.1. Conveying Multi-Sensor Information

#### 5.1.1. Secondary Information Classification

In this subsection, we discuss the performance of the subjects in Tasks FS compared to S. The role of visual feedback (Task VFS) is discussed separately in the following subsection 5.2. Table 3 indicates a statistical difference for the performance in recognizing the medium secondary information class but not for low and high. However, the *post-hoc* test, Table 4, provides more details by showing no significant difference between the performance in Tasks S vs. FS for detecting the medium secondary information class. Therefore, no statistically significant difference is found between conveying two sets of information simultaneously through the single bone conduction channel in the context of recognizing the secondary information compared to conveying one set of information.

#### 5.1.2. Normalized Grasp Force

The Friedman test for the performance of the subjects in the grasp force regulation task as shown in Table 5 does not show any statistically significant difference across the cases of F, FS, and VFS. This is found consistently across the three levels of grasp force. Therefore, no statistically significant reduction in performance is found in the proposed approach against the baseline in the context of grasp force regulation, leading us to conclude that adding a second set of sensor information does not influence the ability to use the first set of sensor information in a closed-loop manner. The standard deviation is qualitatively decreasing for increasing force levels indicating a better repeatability for higher force levels in the case of no visual feedback (F and FS).

It should be noted that the grasping task for F is carried out at the stimulation frequency of 100 Hz, as justified by the psychophysical evaluation. In the case of VFS and FS, the subject also had the chance to carry out the task of regulating grasp force alongside the secondary information classification exercises, which were conducted at [100, 400, 750]Hz. This difference did not significantly influence the ability to control the grasp force.

### 5.2. Role of Visual Feedback

As visual feedback is present in a prosthetic system next to incidental feedback, the influence of visual feedback is investigated while incidental feedback is avoided by using a virtual reality setup. To investigate the influence of visual feedback, whilst feeding back two sets of information, the grasp force has been feed back as a color gradient of the grasped object. Though this is not a real case scenario it contains the same underlying set of information.

#### 5.2.1. Secondary Information Classification

Comparing VFS to S showed a statistically significant increase in the secondary information classification rate in the absence of visual feedback (see Table 4), for the medium secondary information class, but not for low and high. It should be noted that the visual feedback was representing grasp force information and not the secondary information. Comparing VFS to FS does not yield any statistically significant difference in performance (see Tables 3, 4). Several explanations are possible. It could suggest that the subjects were able to learn the meaning of the feedback and perform better or that the reduced cognitive effort increased performance. However, the data collected in this study did not permit the authors to draw further conclusions.

#### 5.2.2. Normalized Grasp Force

The obtained normalized grasp force performance shows no statistically significant difference between the tasks involving visual feedback VFS compared to those with no visual feedback (F and FS). A smaller variance of the normalized grasp force is obtained for VFS compared to F and FS. It should be noted that VFS adds visual feedback for the same sensory information, namely the grasp force. A similar observation was reported in Patterson and Katz (1992) stating that the primary advantage of supplemental feedback is to reduce the variability of responses. This decrease can not be observed for F compared to FS as it does not add more feedback of the same sensory information but rather superimposes other types of sensory information.

It should be noted that the results are obtained using a virtual reality setup. This allows the control of the provision of visual feedback while guiding the subjects through the grasp task experiment. Admittedly it abstracts the experiment from a practical grasping task. However, it does not take away the main premise from the study, which is to understand how well two sets of information can be conveyed in this novel manner.

## 6. Conclusion

This study investigated the efficacy of conveying multi-sensor information via fewer feedback channels in a prosthetics context. Two sets of sensor information: grasp force and a secondary information, are conveyed simultaneously to human users through one feedback channel (a vibrotactile transducer on bone conduction). Human subject experiment was conducted using physical vibrotactile transducers on the elbow bony landmark and virtual reality environment to simulate the prosthetic grasping force regulation and secondary information classification tasks. It was found that the subjects were able to discern the two sets of feedback information, sufficient to perform the grasping and secondary information classification tasks to a performance not inferior to that when carried out with only one set of feedback information. The addition of visual feedback, a common feedback mechanism present in prostheses, was found to improve the repeatability of grasp force regulation as reported in literature.

It is expected that the result is generalizable to other types of information and modalities (not limited to grasp force and bone conduction stimulation) and more freedom in the selection of the number of independent sets of sensor information *N* and feedback stimulation channel *M*, as long as *N* > *M*. The second set of information was generalized and labeled secondary information but can be multiple in a real world application e.g., temperature, friction.

It should be noted that in this experiment, one set of sensor information was used explicitly in the closed-loop performance of grasp force regulation, while the other set constitutes additional information. Future work will investigate other modulation techniques to encode the multi-sets of information into the one feedback stimulation channel and algorithms to find an optimal matching between sensory information and provided feedback.

## Data Availability Statement

The datasets generated for this study are available on request to the corresponding author.

## Ethics Statement

The studies involving human participants were reviewed and approved by Ethics Committee of the University of Melbourne. Project numbers are 1852875.2 and 1750711.1. The patients/participants provided their written informed consent to participate in this study.

## Author Contributions

RM, RG-R, AM, YT, and DO: literature, experiment, data analysis, and paper. GA and PC: paper design, experiment design, and paper review.

## Conflict of Interest

The authors declare that the research was conducted in the absence of any commercial or financial relationships that could be construed as a potential conflict of interest.
